# Imported Tungiasis in Greece: Secondary Household Transmission and Transient Mixed Liver Enzyme Elevation

**DOI:** 10.3390/tropicalmed11060169

**Published:** 2026-06-21

**Authors:** Thomas Fotas, Ioannis A. Giantsis, Menelaos Lefkaditis, Ioannis S. Pappas, Mathis A. B. Christodoulopoulos, Efterpi Zafiriou, Electra Nicolaidou, Alexander C. Katoulis, Georgios Christodoulopoulos

**Affiliations:** 1 Department of Dermatology and Venereology, General Hospital “Asklepieio Voulas”, 16673 Voula, Greece; thomasfotas@uth.gr; 2 Department of Animal Science, Faculty of Agriculture, Forestry and Natural Environment, Aristotle University of Thessaloniki, 54124 Thessaloniki, Greece; igiants@auth.gr; 3 Laboratory of Microbiology and Parasitology, Faculty of Veterinary Science, University of Thessaly, 43100 Karditsa, Greece; mlefkaditis@vet.uth.gr; 4 Laboratory of Pharmacology and Toxicology, Faculty of Veterinary Science, University of Thessaly, 43100 Karditsa, Greece; ipappas@vet.uth.gr; 5 Faculté des Sciences, Site St Charles, Aix Marseille Université, 13331 Marseille, France; mathis.christodoulopoulos@etu.univ-amu.fr; 6 Department of Dermatology, Faculty of Medicine, School of Health Sciences, University of Thessaly, Biopolis, 41500 Larissa, Greece; zafevi@med.uth.gr; 7 First Department of Dermatology and Venereology, “A. Sygros” Hospital for Skin and Venereal Diseases, School of Medicine, National and Kapodistrian University of Athens, 11528 Athens, Greece; electra.nicol@gmail.com; 8 Second Department of Dermatology and Venereology, “Attikon” General University Hospital, School of Medicine, National and Kapodistrian University of Athens, 12461 Athens, Greece; alexanderkatoulis@yahoo.co.uk; 9 Department of Animal Science, Agricultural University of Athens, Iera Odos 75 Str., 11855 Athens, Greece

**Keywords:** *Tunga penetrans*, travel-associated infection, household transmission, dermoscopy, molecular identification, sub-Saharan Africa, mediterranean region, zoonotic parasites, liver enzyme abnormalities

## Abstract

Tungiasis is a cutaneous ectoparasitosis caused by the penetration of gravid female *Tunga penetrans* fleas into the epidermis. Although endemic in tropical and subtropical regions, it remains rare in Europe, where most cases are travel-associated and secondary household transmission is seldom documented. This study describes imported tungiasis in Greece and investigates possible secondary household transmission in a non-endemic setting. Seven Greek men residing in Attica developed tungiasis following occupational exposure in Tanzania, together with one secondary case in a non-travelling household contact who had never travelled outside Greece. Diagnosis was based on clinical and dermoscopic findings and confirmed by amplification and sequencing of the mitochondrial cytochrome oxidase I (COI) gene. Household investigations were also performed. Eight male patients presented with painful plantar and/or subungual nodular lesions. Sequence analysis of COI demonstrated 657/662 bp (99%) identity with the *Tunga penetrans* reference sequence, and identical sequences were identified in all samples. A representative sequence was deposited in GenBank (accession no. PZ336383). All patients exhibited mild-to-moderate elevations of hepatocellular and cholestatic liver enzymes, which resolved within two weeks following treatment. One probable secondary household case was identified, and no infestation was detected among additional cohabitants or companion animals. This report documents imported tungiasis with probable secondary household transmission in Greece and highlights the importance of clinical awareness and environmental assessment in non-endemic settings.

## 1. Introduction

Tungiasis is a cutaneous ectoparasitosis caused predominantly by *Tunga penetrans*, although human infestation with *Tunga trimamillata* has also been reported in Latin America [1,2,3,4]. The disease is rare in Europe and North America but remains highly endemic in parts of sub-Saharan Africa, the Caribbean, and South America, where it constitutes a significant public health problem [5,6,7].

Infection is typically acquired through direct contact with infested soil while walking barefoot, particularly in peri-domestic areas and on unpaved roads where the off-host developmental stages of *Tunga penetrans* can survive and persist [2,6,8]. Adult female fleas spend little time on the host surface before rapidly penetrating the skin. Following penetration, only the posterior end of the abdomen remains exposed through a small cutaneous opening, through which the flea respires, copulates, and releases eggs into the environment [5,6,9].

Clinically, lesions are commonly located in the periungual and interdigital regions of the feet. Once embedded in the skin, the flea undergoes marked hypertrophy (neosomy), increasing its volume by approximately 2000–3000-fold [9,10]. The parasite induces a localized inflammatory response, resulting in painful nodular lesions that are frequently complicated by secondary bacterial infection. In endemic settings, repeated infestations may lead to substantial morbidity, including chronic ulceration, deformity, and impaired mobility [5].

In non-endemic regions such as Europe and North America, tungiasis is primarily diagnosed in travelers returning from tropical and subtropical areas. Reliable data on its true prevalence among travelers remain limited, as only a proportion of affected individuals seek medical attention or are referred to specialized centers. Nevertheless, available studies suggest that tungiasis is not uncommon among returning travelers. An airport-based survey of international tourists departing from Brazil reported a tungiasis prevalence of 3.2% [11], while a prospective study of 269 patients presenting with travel-associated dermatoses at a hospital in Paris identified tungiasis in 6% of cases [12].

The epidemiology of tungiasis reflects a complex interaction between human hosts, animal reservoirs, and environmental factors. In endemic settings, animals can play a role in maintaining the parasite life cycle and sustaining environmental contamination but are not essential. The cycle can be maintained without any animal reservoir involvement [6,13]. The off-host developmental stages of *Tunga penetrans* occur in soil and are highly dependent on environmental conditions, including temperature, relative humidity, and substrate characteristics, which influence the survival and development of eggs, larvae, and pupae. Optimal development is generally associated with warm temperatures and moderate humidity, whereas excessive dryness or extreme heat may impair larval survival and disrupt the life cycle [6,14]. These ecological dynamics remain poorly characterized in non-endemic regions, where climatic and environmental conditions may limit sustained transmission.

Here, we report a case series of imported tungiasis in seven Greek men following occupational exposure in Tanzania, with subsequent involvement of a non-traveling household contact. This study highlights the potential for intra-household transmission in a non-endemic setting, underscores the importance of detailed travel history, clinical recognition, and dermoscopic evaluation, and describes the associated clinical and biochemical findings. In addition, environmental and household investigations, including assessment of companion animals and local climatic conditions, were conducted to explore possible transmission pathways. Molecular characterization of the isolated fleas through analysis of the mitochondrial cytochrome oxidase I (COI) gene further contributes to the limited molecular data available from imported cases in Europe.

## 2. Materials and Methods

### 2.1. Patients and Clinical Evaluation

The present case series included seven male patients who presented to the Department of Dermatology and Venereology, General Hospital “Asklepieio Voulas”, Athens, Greece, with painful foot lesions compatible with tungiasis. All had worked in construction in the Moshi area of northeastern Tanzania, close to the Kenyan border, for approximately six months before returning to Greece in early June 2023. Two additional co-workers returned at the same time but did not report any symptoms and therefore did not present to the hospital for clinical evaluation.

Among the seven male patients, one 51 year old man cohabited with two non-traveling adult male sexual partners (aged 42 and 40 years) in the same household. The 42-year-old cohabiting sexual partner was subsequently evaluated after developing a compatible lesion approximately 30 to 35 days following the patient’s return.

In total, eight male patients were included in the study. Clinical evaluation comprised detailed travel history, physical examination, and dermoscopic assessment. Dermoscopy was performed using a handheld dermoscope (DermLite DL4, 3Gen Inc., San Juan Capistrano, CA, USA) at ×10 magnification under both polarized and non-polarized illumination, in accordance with established dermoscopic criteria for tungiasis [6,15].

The temporal sequence of clinical events and interventions is summarized in Table 1.

### 2.2. Laboratory Investigations

All patients underwent routine laboratory testing at presentation, including complete blood count (CBC) and serum biochemical analysis. Hematological parameters were measured using an automated hematology analyzer (Sysmex XN-1000, Sysmex Corporation, Kobe, Japan). Biochemical analyses, including liver function tests, aspartate aminotransferase (AST), alanine aminotransferase (ALT), alkaline phosphatase (ALP), and gamma-glutamyl transferase (γGT), were performed using an automated chemistry analyzer (Cobas 8000 modular analyzer series, Roche Diagnostics, Mannheim, Germany), following the manufacturer’s standardized protocols.

Quality control procedures were performed daily using commercial control sera to ensure analytical accuracy. Follow-up laboratory testing was conducted 14 days after treatment to assess normalization of biochemical parameters.

### 2.3. Household Investigation

Household investigations were conducted for patients reporting residence in homes with outdoor areas (yards or surrounding land) and/or the presence of companion animals. These households were visited, and the domestic environment was assessed observationally, including the characteristics of indoor and outdoor spaces, the nature of ground and flooring surfaces, peri-domestic conditions, and the access of companion animals to these areas. No standardized environmental sampling protocol was applied, and no soil or dust samples were collected or analyzed for the presence of *Tunga penetrans* developmental stages.

Meteorological data were derived from publicly available regional datasets and are presented as representative values for coastal Attica during the study period.

Cohabiting household members were clinically assessed at their residence on two occasions, 14 days apart, during July–August 2023 by a qualified dermatologist (T.F.). Companion animals were examined by an experienced veterinarian (G.C.) for the presence of ectoparasitic lesions consistent with tungiasis or other infestations. The first household visit was carried out after environmental control measures had reportedly been implemented by the occupants.

### 2.4. Treatment and Follow-Up

Following clinical assessment, all patients underwent surgical extraction of the embedded parasites as part of routine clinical management. The procedure was performed under aseptic conditions and local anaesthesia (lidocaine HCl 1%) and involved careful enlargement of the central opening using a sterile needle or scalpel, followed by complete removal of the parasite and surrounding necrotic tissue, with care taken to avoid rupture and secondary inflammation. Parasite material obtained during extraction was collected under sterile conditions and preserved in 70% ethanol until molecular analysis.

Topical ivermectin (1% cream; Soolantra^®^, Galderma International SAS, Paris, France) was applied locally following extraction. In addition, a single oral dose of ivermectin (200 μg/kg body weight) was administered as adjunctive therapy, in accordance with previously reported management approaches [6,16].

Environmental control measures were implemented, according to the patients’ reports, including targeted spraying of household and yard areas with a 0.05% phoxim solution (Sebacil^®^, Bayer Animal Health GmbH, Leverkusen, Germany) [17,18,19]. In households with companion animals, topical ivermectin (0.5% pour-on formulation) was applied percutaneously at a dose of 500 μg/kg body weight (1 mL/10 kg) [20].

Clinical follow-up of the patients was performed at 7 and 14 days post-treatment.

### 2.5. Molecular Analysis

For molecular characterization, one embedded flea from each patient was selected for analysis. When multiple lesions were present, the most intact recovered specimen was preferentially chosen; otherwise, a representative specimen was selected.

Genomic DNA was extracted from the selected specimen using a phenol–chloroform protocol as described by Sambrook and Russell [21], with minor modifications. DNA concentration and purity were assessed using a NanoDrop 2000 spectrophotometer (Thermo Fisher Scientific, Waltham, MA, USA), and DNA integrity was verified by agarose gel electrophoresis.

A fragment of the mitochondrial cytochrome oxidase I (COI) gene was amplified by polymerase chain reaction (PCR) using the universal primers LCO1490 (5′-GGTCAACAAATCATAAAGATATTGG-3′) and HCO2198 (5′-TAAACTTCAGGGTGACCAAAAAATCA-3′), as originally described by Folmer et al. [22]. PCRs were performed in a total volume of 25 μL containing 1× PCR buffer, 2.0 mM MgCl_2_, 200 μM of each dNTP, 0.4 μM of each primer, 1 U Taq DNA polymerase (Invitrogen, Carlsbad, CA, USA), and 50–100 ng of template DNA.

Thermal cycling conditions included an initial denaturation at 94 °C for 5 min, followed by 35 cycles of denaturation at 94 °C for 30 s, annealing at 50 °C for 30 s, and extension at 72 °C for 1 min, with a final extension at 72 °C for 7 min.

PCR products were separated on a 1.5% agarose gel stained with ethidium bromide and visualized under UV illumination. Amplicons were purified using a commercial PCR purification kit (Qiagen, Hilden, Germany) and sequenced bidirectionally (Eurofins Genomics, Ebersberg, Germany). Forward and reverse sequences were assembled and manually edited using BioEdit software (version 7.2.5).

Species identification was performed using the BLAST algorithm (National Center for Biotechnology Information, Bethesda, MD, USA; https://blast.ncbi.nlm.nih.gov) against the GenBank database.

### 2.6. Ethical Considerations

The study was conducted in accordance with the Declaration of Helsinki. Written informed consent was obtained from all patients for diagnostic procedures, molecular analysis, and publication of anonymized clinical data and images. According to institutional policy, formal ethics committee approval was not required for this case series.

## 3. Results

**Figure 1:**
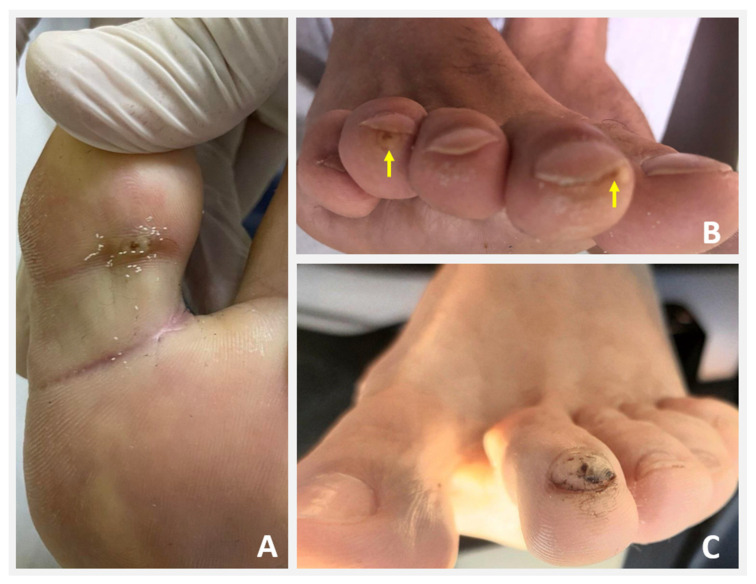
Clinical presentation of tungiasis lesions on the feet. (**A**) Single plantar lesion on the left hallux, with parasite eggs visible on the adjacent skin surface. (**B**) Periungual lesions affecting the 2nd and 4th toes of the right foot (yellow arrows). (**C**) Subungual lesion on the 2nd toe of the left foot.

All eight patients, including seven men with recent travel history to Tanzania and one non-traveling household contact, presented with painful plantar and/or subungual nodular lesions. The patients commonly reported moderate pruritus and a sensation of a foreign body in the affected area. Clinically, the lesions were typically yellowish and characterized by a central dark punctum surrounded by a mildly erythematous halo (Figure 1A–C). No lower limb edema or regional lymph node enlargement was detected during clinical examination.

**Figure 2:**
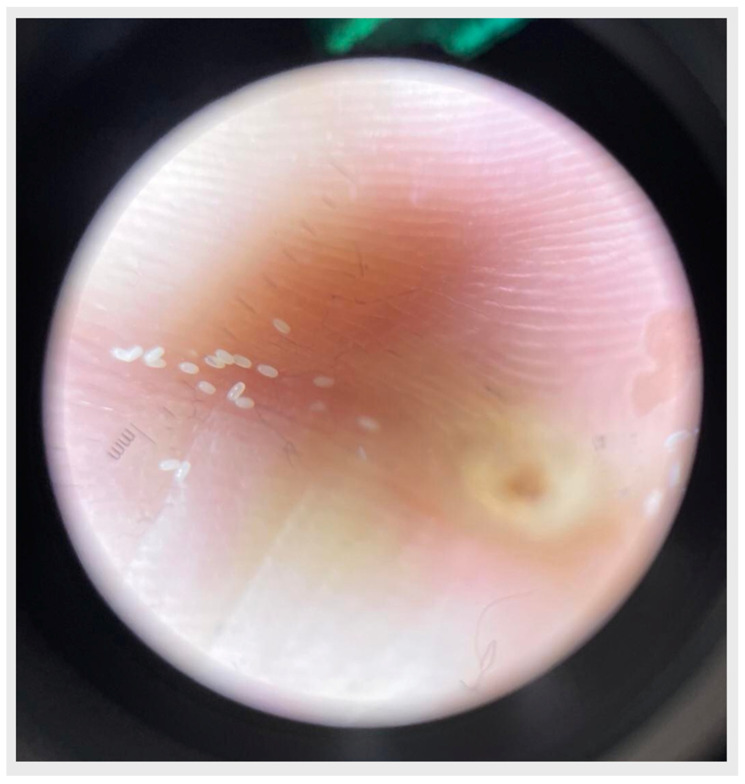
Dermoscopic appearance of a tungiasis lesion. A central brown crateriform structure is visible, surrounded by a peripheral yellowish halo and a distinct pigmented ring. Several parasite eggs are attached to the adjacent plantar skin surface.

Dermoscopy consistently revealed a central brown crateriform structure surrounded by a peripheral yellowish halo and a distinct pigmented ring. In several lesions, multiple whitish ovoid structures corresponding to parasite eggs were identified, further supporting the clinical diagnosis of tungiasis (Figure 2). Notably, in patients who had been wearing socks prior to clinical evaluation, a more pronounced accumulation of parasite eggs on the skin surface of the feet was observed. In our clinical experience, this finding facilitated both suspicion and confirmation of the diagnosis.

In the non-traveling household contact, a single lesion was identified in the subungual region of the second toe of the left foot (Figure 1C). The absence of travel history, together with compatible clinical and dermoscopic findings, suggests probable secondary household transmission.

Across the case series, lesions were confined to the feet, predominantly affecting plantar and periungual regions. The number and anatomical distribution of lesions per patient are summarized in Table 2.

Routine laboratory testing revealed mild-to-moderate elevations of liver enzymes in all patients, involving both hepatocellular (AST, ALT) and cholestatic (ALP, γGT) parameters, without a consistent biochemical pattern. Complete blood count findings and other biochemical parameters remained within reference ranges. Detailed liver enzyme findings at presentation and follow-up are presented in Table 3. Notably, none of the patients exhibited fever or leukocytosis.

Clinical follow-up at 7 and 14 days post-treatment demonstrated uncomplicated healing of the lesions, with no evidence of new lesions or secondary complications. Laboratory reassessment at 14 days confirmed normalization of the previously elevated liver enzyme levels (Table 3).

### 3.1. Molecular Findings

Eight samples, one from each patient, were subjected to molecular analysis. PCR amplification of the mitochondrial cytochrome oxidase I (COI) gene yielded a single amplicon of the expected size in all analyzed samples, while negative controls showed no amplification.

BLAST analysis demonstrated 657/662 bp (99%) nucleotide identity with the *Tunga penetrans* reference sequence (GenBank accession no. PV426769), confirming species identification. Identical sequences were obtained from all samples, suggesting a common source of infestation.

The representative sequence was deposited in GenBank under accession number PZ336383.

### 3.2. Household Investigation

Two of the seven male patients resided in houses with outdoor areas (backyards), including the patient associated with the probable secondary household transmission event. Both households also kept companion animals.

The two households were in coastal areas of Attica. One was situated in the seaside area of Megara (western Attica), while the other, associated with the probable secondary transmission event, was in Porto Rafti, an Aegean coastal settlement in Eastern Attica. Both settings were characterized by peri-domestic environments with direct contact between indoor and outdoor spaces. Environmental conditions during the study period (June to July 2023) were typical of the Mediterranean summer, with high ambient temperatures, moderate relative humidity, and minimal precipitation. Detailed meteorological data are presented in Table 4.

The first household consisted of a couple (the patient and his spouse) and two children aged 5 and 7 years and kept a single dog with unrestricted access to both indoor and outdoor areas. The second household consisted of the patient and two adult male cohabitants and kept one dog and two cats, all of which had unrestricted access to both indoor and outdoor areas.

Both residences were modern houses of recent construction, built primarily using concrete and cement, with cemented flooring throughout the indoor living areas. Each property included a surrounding outdoor area, part of which was landscaped and maintained as a flower garden.

During the two household assessments, no additional cases of tungiasis were identified among the spouse, two children, or the remaining adult cohabitant. Likewise, none of the two dogs or two cats showed clinical evidence of tungiasis or any other ectoparasitic infestation.

### 3.3. Treatment Outcome and Follow-Up

All patients underwent surgical extraction of the embedded parasites. Clinical follow-up demonstrated uncomplicated healing of the lesions, with no evidence of secondary infection or recurrence.

Liver enzyme abnormalities were transient, with all measured parameters returning to within reference ranges within two weeks following treatment (Table 3).

## 4. Discussion

Tungiasis remains an uncommon but increasingly recognized travel-associated dermatosis in non-endemic regions such as Europe [23,24,25,26,27,28]. In this context, the present case series highlights not only the occurrence of imported infection following travel to Tanzania, but also the potential for secondary household transmission under specific environmental conditions. These findings contribute to the understanding of the epidemiology and transmission dynamics of tungiasis outside endemic settings.

In non-endemic regions, tungiasis poses significant diagnostic challenges due to its rarity and the consequently low level of clinical suspicion, often leading to misdiagnosis as a bacterial infection, wart, or foreign body [6,16,25]. The differential diagnosis also includes clavus (corn), pyogenic granuloma, subungual hematoma, paronychia, myiasis, cutaneous larva migrans, arthropod bites, epidermoid cysts, and retained foreign bodies. Careful clinical examination, a detailed travel history, and dermoscopy are therefore essential for establishing the correct diagnosis and distinguishing tungiasis from these conditions [6,8].

In the present series, the clinical manifestations were consistent with previous reports, with lesions predominantly localized to plantar and periungual regions and characterized by nodular morphology, a central punctum, and surrounding inflammation. Dermoscopy substantially improved diagnostic accuracy by enabling visualization of characteristic features such as the central pore, pigmented ring, and whitish ovoid structures corresponding to parasite eggs, which are considered diagnostic hallmarks [5,6,29]. The consistent dermoscopic findings observed in our cases further support its value as a rapid and non-invasive diagnostic tool in non-endemic settings. In our study, the most prominent clinical feature of the infestation was the presence of parasite eggs, with a notably increased accumulation on the skin of the feet among patients who were wearing socks prior to clinical examination, a finding that facilitated clinical recognition. As illustrated in Figure 1A and Figure 2, the eggs appeared as small, oval, whitish to translucent structures clustered on the skin adjacent to the lesion.

This study further expands the epidemiological understanding of tungiasis in Europe by documenting probable secondary household transmission. The classical transmission cycle of *Tunga penetrans* involves contamination of the environment with eggs expelled by embedded fleas, followed by development of the off-host stages in the soil and subsequent infestation of susceptible hosts under favorable conditions. While this transmission pathway is well established in endemic settings, it has not, to our knowledge, been documented in non-endemic regions, where most reported cases remain travel-associated [6,8,12]. Rare instances of direct inter-host transfer of adult female fleas between individuals in close proximity have also been described. These reports involve free-living female fleas actively seeking a penetration site [30]. In the present case, the index patient and the subsequently affected individual were cohabiting sexual partners, and their close physical contact could theoretically have facilitated transfer of a free-living flea. Nevertheless, this explanation appears less likely because the index patient had already returned from Tanzania before contact with his partner in Greece, and the intervening travel period would have provided sufficient time for any infective flea acquired abroad to penetrate the skin of the index patient. Furthermore, direct inter-host transfer of adult female fleas appears to be a rare event and has been reported primarily in the setting of heavy infestations. In the present case series, all patients harbored only one to three lesions (Table 2), making this mechanism less likely. Therefore, the temporal relationship between returning traveler and lesion development in the non-traveling cohabiting partner, together with the low parasite burden observed, is more compatible with environmental contamination being the most plausible mechanism of secondary transmission, although direct inter-host transfer cannot be completely excluded.

Environmental conditions are key determinants of the off-host development of *Tunga penetrans*. The life cycle depends on warm temperatures and adequate soil humidity, with optimal development generally occurring at approximately 25–31 °C and moderate moisture levels that prevent desiccation of eggs and larvae [6,31]. Conversely, excessively dry conditions or extreme temperatures can impair the survival of immature stages and interrupt the life cycle [12,32].

In the context of global climate change, there has been increasing interest in whether shifts in temperature and precipitation patterns could modify the geographical distribution and seasonal activity of arthropod vectors and ectoparasites. Although it is premature to predict the establishment of *Tunga penetrans* in currently non-endemic regions, warmer climatic conditions and the occurrence of favorable microhabitats could theoretically increase opportunities for limited local transmission following imported cases. Nevertheless, the successful establishment of the parasite is likely to depend on a complex interplay of climatic, environmental, ecological, socioeconomic, and host-related factors rather than climate alone [33,34,35].

In the present study, the household associated with secondary transmission was in a coastal area of Attica (Porto Rafti), where climatic conditions during June–July 2023 (Table 4) were characterized by high temperatures, moderate relative humidity, and minimal precipitation, including a heatwave period with temperatures exceeding 40 °C. These conditions may have been partially permissive for limited off-host development, particularly in sheltered peri-domestic microhabitats. However, extreme temperatures and reduced soil moisture likely constrained larval survival, limiting the establishment of a sustained environmental reservoir. In addition, the timely implementation of control measures, including the application of environmental antiparasitic spray and prophylactic treatment of companion animals, may have further reduced environmental contamination and interrupted the parasite life cycle. Accordingly, the combined effect of these environmental constraints and intervention measures may explain the occurrence of only a single secondary case, despite close and prolonged cohabitation among household members with comparable levels of environmental exposure, including shared use of living spaces and frequent contact with indoor surfaces. At the same time, phoxim should be used judiciously and in accordance with applicable regulations and manufacturer recommendations because of its potential environmental toxicity and adverse effects on non-target organisms [36].

The household investigation provides additional insight into human–animal–parasite interactions in a non-endemic setting. Despite the presence of companion animals in two households, none of the examined pets (two dogs and two cats) showed clinical evidence of tungiasis. This finding is noteworthy, given that dogs, pigs, and rodents are recognized reservoirs in endemic regions [6,13]. The absence of infestation in pets, despite the lack of prior ectoparasite prophylaxis, suggests that under the observed environmental conditions, companion animals may not have played a significant role as hosts.

Behavioral factors, such as grooming habits and patterns of contact with infested substrates, may also influence host susceptibility. Host preference may vary according to ecological context, with humans potentially acting as more exposed hosts in peri-domestic environments in non-endemic areas [2]. Although these interpretations remain speculative due to the small sample size, they highlight the complexity of transmission dynamics outside endemic regions.

Management in all cases consisted of surgical extraction combined with topical and systemic ivermectin, resulting in resolution without recurrence. Surgical removal remains the standard treatment for patients with a limited lesion burden and, when performed carefully, is generally sufficient to achieve cure [6,16]. In the present case series, adjunctive ivermectin was administered as part of the treating physicians’ clinical management, although its additional benefit following complete parasite extraction remains uncertain.

Molecular confirmation using COI gene sequencing further strengthened diagnostic accuracy. One embedded flea from each patient was selected for molecular analysis, and all eight analyzed specimens yielded identical COI sequences. The obtained sequence demonstrated 657/662 bp (99%) identity with the available *Tunga penetrans* reference sequence (GenBank accession no. PV426769), supporting species identification. The observed five nucleotide differences relative to the available GenBank reference sequence were considered compatible with identification of the specimens as *Tunga penetrans*.

Although the identical sequences are consistent with a common origin of the analyzed fleas, they do not by themselves distinguish between acquisition from a shared source in Tanzania and subsequent local transmission. The limited availability of publicly accessible COI sequence data for *Tunga penetrans* currently precludes meaningful phylogenetic or haplotype comparisons, although such analyses may become feasible as additional sequence resources become available. Notably, the recent publication of the first complete mitochondrial genome of *Tunga penetrans* is expected to facilitate future molecular epidemiological and phylogenetic studies of this species [37]. The COI gene was selected as the standard DNA barcode marker for arthropod species identification owing to its high interspecific variability and widespread use in molecular taxonomy. Recent molecular studies have also highlighted the value of DNA-based approaches for the identification of *Tunga penetrans* and its developmental stages [22,38,39,40]. Although COI data for *Tunga trimamillata* are not currently available in GenBank, differentiation from this species was not considered essential given the epidemiological context of exposure in Tanzania, where *Tunga penetrans* is endemic, whereas *Tunga trimamillata* has been reported primarily in Latin America.

An additional finding of interest was the presence of mild-to-moderate elevations of both hepatocellular and cholestatic liver enzymes in all patients (Table 2). Given that tungiasis is a strictly cutaneous ectoparasitosis without known hepatic tropism, direct liver involvement appears unlikely. One possible explanation is a transient systemic inflammatory response, as pro-inflammatory cytokines, including interleukin-6 and tumor necrosis factor α, are known to influence hepatocellular function and bile secretion [41,42]. Similar transient enzyme elevations have been described in inflammatory conditions without primary liver disease [43]. The rapid normalization of liver enzyme levels following parasite removal is compatible with a reversible functional disturbance. However, this interpretation should be considered hypothesis-generating rather than conclusive. The observational nature of this case series and the absence of additional investigations, such as inflammatory biomarkers or systematic evaluation for alternative causes of liver enzyme abnormalities, preclude establishment of a causal relationship between tungiasis and biochemical findings. Other potential causes of transient liver enzyme abnormalities, including concomitant viral infections, alcohol consumption, recent medication use, metabolic dysfunction-associated steatotic liver disease, occult hepatobiliary disorders, or other unrelated inflammatory conditions, cannot be entirely excluded in the absence of a comprehensive diagnostic work-up [43,44,45]. To our knowledge, there is currently no evidence that tungiasis is associated with chronic hepatic injury or long-term sequelae such as chronic hepatitis, liver fibrosis, cirrhosis, or hepatocellular carcinoma. The biochemical abnormalities observed in the present study appear to have been transient and reversible, and further studies are required to determine whether they represent an incidental finding or an underrecognized aspect of the host response to infestation.

Overall, this report underscores the importance of considering tungiasis in the differential diagnosis of nodular or subungual lesions in travelers returning from endemic regions. It also highlights the need to examine close household contacts and to consider environmental factors when evaluating potential secondary transmission.

## 5. Conclusions

Imported tungiasis remains an uncommon but clinically relevant parasitosis in Europe. This case series documents imported tungiasis acquired in Tanzania and provides evidence compatible with probable secondary household transmission in Greece. Molecular identification confirmed the species as *Tunga penetrans*, and the epidemiological findings support the possibility of local household transmission in a non-endemic setting.

In non-endemic settings, timely diagnosis relies on clinical awareness, detailed travel history, and the use of dermoscopy. Environmental conditions may permit limited local transmission under favorable circumstances, while timely intervention measures, including environmental disinfection and antiparasitic treatment of companion animals, may help reduce environmental contamination and interrupt the parasite life cycle.

Preventive measures, including the use of protective footwear and attention to environmental hygiene, remain essential. Further studies are needed to clarify the relative contributions of environmental factors, host-related characteristics, and intervention measures, including the role of domestic animals, in shaping transmission dynamics outside endemic areas.

## Figures and Tables

**Table 1:** Timeline of key clinical and environmental interventions in the case series.

Date	Event
3 June 2023	Return of seven workers from Tanzania to Greece
6–8 June 2023	Seven patients presented to the hospital, underwent clinical evaluation, and received treatment
13–17 June 2023	Application of antiparasitic spray in household environments, as reported by the patients
20–23 June 2023	Clinical follow-up of the seven patients at the hospital
10 July 2023	Presentation, clinical evaluation, and treatment of the eighth case (non-traveling household contact)
14 July 2023	First household visit to premises with outdoor areas; clinical assessment and antiparasitic treatment of companion animals
15 July 2023	Re-application of antiparasitic spray in the visited household environment
25 July 2023	Clinical follow-up of the eighth patient at the hospital
28 July 2023	Second household visit (follow-up) to premises with outdoor areas

**Table 2:** Distribution and number of lesions in male patients with imported tungiasis.

Patient	Age (Years)	Travel History	Number of Lesions	Anatomical Location(s)
P1	55	Yes (Tanzania)	3	Left hallux (plantar); right foot, 2nd and 4th toes (subungual)
P2	60	Yes (Tanzania)	2	Plantar surface, right foot
P3	51	Yes (Tanzania)	1	Left hallux (subungual)
P4	18	Yes (Tanzania)	2	Plantar region, bilateral feet
P5	21	Yes (Tanzania)	1	Right foot, periungual region
P6	45	Yes (Tanzania)	2	Left foot, plantar and periungual regions
P7	64	Yes (Tanzania)	1	Right hallux (subungual)
P8 *	42	No	1	Left foot, 2nd toe (subungual)

* P8 represents the non-traveling household contact.

**Table 3:** Liver Enzyme Findings in Patients with Tungiasis at Presentation and Follow-up.

Patient	AST (U/L)		ALT (U/L)		ALP (U/L)		γGT (U/L)	
	Pre	Post	Pre	Post	Pre	Post	Pre	Post
P1	82	32	96	38	165	114	88	42
P2	68	30	74	35	140	105	52	40
P3	45	28	62	33	120	98	40	35
P4	95	36	110	62	180	120	102	50
P5	38	30	48	34	155	108	60	38
P6	72	34	85	37	135	102	70	41
P7	55	29	60	32	125	100	45	36
P8	50	27	58	34	115	110	85	35
Mean (SD)	63 (20)	31 (3)	74 (21)	38 (10)	142 (23)	107 (7)	68 (22)	40 (5)

Abbreviations: AST, aspartate aminotransferase; ALT, alanine aminotransferase; ALP, alkaline phosphatase; γGT, gamma-glutamyl transferase. Reference ranges (adults): AST: 10–40 U/L; ALT: 7–56 U/L; ALP: 44–147 U/L; γGT: 9–48 U/L. Note: Pre = at presentation; Post = 14 days after treatment.

**Table 4:** Mean temperature and relative humidity in coastal Attica (Megara and Porto Rafti) during June–July 2023.

Month	Mean Temperature (°C)	Temperature Range (°C)	Mean Relative Humidity (%)	Humidity Range (%)	Precipitation (mm)
June 2023	28–30	20–34	50–55	35–70	~10
July 2023	31–33	24–42	40–50	25–65	~5–8

Source: Hellenic National Meteorological Service (HNMS) and National Observatory of Athens (meteo.gr), representative data for coastal Attica.

## Data Availability

Data is contained within the article.

## Abbreviations

The following abbreviations are used in this manuscript:

COI	Mitochondrial cytochrome oxidase I
AST	Aspartate aminotransferase
ALT	Alanine aminotransferase
ALP	Alkaline phosphatase
γGT	Gamma-glutamyl transferase
